# Cognitive impairment in post-acute COVID-19 syndrome: a scoping review

**DOI:** 10.1055/s-0043-1777115

**Published:** 2023-12-29

**Authors:** Gabriela Cabett Cipolli, Vanessa Alonso, Clarissa Lin Yasuda, Daniela de Assumpção, Meire Cachioni, Ruth Caldeira de Melo, Kathryn Hinsliff-Smith, Mônica Sanches Yassuda

**Affiliations:** 1 Universidade Estadual de Campinas, Faculdade de Ciências Médicas, Programa de Pós-graduação em Gerontologia, Campinas SP, Brazil.; 2 Universidade Estadual de Campinas, Faculdade de Ciências Médicas, Departamento de Neurologia, Campinas SP, Brazil.; 3 Universidade de São Paulo, Escola de Artes, Ciências e Humanidades, Programa de Pós-graduação em Gerontologia, São Paulo SP, Brazil.; 4 De Montfort University, Faculty of Health and Life Sciences, Leicester School of Nursing & Midwifery, United Kingdom.

**Keywords:** Cognitive Dysfunction, Memory, Episodic, Attention, SARS-CoV-2, Post-Acute COVID-19 Syndrome, Disfunção Cognitiva, Memória Episódica, Atenção, SARS-CoV-2, Síndrome Pós-COVID-19 Aguda

## Abstract

Emerging studies indicate the persistence of symptoms beyond the acute phase of COVID-19. Cognitive impairment has been observed in certain individuals for months following infection. Currently, there is limited knowledge about the specific cognitive domains that undergo alterations during the post-acute COVID-19 syndrome and the potential impact of disease severity on cognition. The aim of this review is to examine studies that have reported cognitive impairment in post-acute COVID-19, categorizing them into subacute and chronic phases. The methodology proposed by JBI was followed in this study. The included studies were published between December 2019 and December 2022. The search was conducted in PubMed, PubMed PMC, BVS – BIREME, Embase, SCOPUS, Cochrane, Web of Science, Proquest, PsycInfo, and EBSCOHost. Data extraction included specific details about the population, concepts, context, and key findings or recommendations relevant to the review objectives. A total of 7,540 records were identified and examined, and 47 articles were included. The cognitive domains most frequently reported as altered 4 to 12 weeks after acute COVID-19 were language, episodic memory, and executive function, and after 12 weeks, the domains most affected were attention, episodic memory, and executive function. The results of this scoping review highlight that adults with post-acute COVID-19 syndrome may have impairment in specific cognitive domains.

## Resumo

Estudos emergentes indicam a persistência dos sintomas além da fase aguda da COVID-19. O comprometimento cognitivo foi observado em alguns indivíduos durante meses após a infecção. Atualmente, há pouco conhecimento sobre os domínios cognitivos específicos que sofrem alterações durante a síndrome pós-aguda da COVID-19 e o possível impacto da gravidade da doença na cognição. O objetivo desta revisão é examinar estudos que relataram comprometimento cognitivo na COVID-19 pós-aguda, categorizando-os em fases subaguda e crônica. A metodologia proposta pela Joanna Briggs Institute foi seguida neste estudo. Os estudos incluídos foram publicados entre dezembro de 2019 e dezembro de 2022. A busca foi realizada no PubMed, PubMed PMC, BVS – BIREME, Embase, SCOPUS, Cochrane, Web of Science, Proquest, PsycInfo e EBSCOHost. A extração de dados incluiu detalhes específicos sobre a população, os conceitos, o contexto e as principais descobertas ou recomendações relevantes para os objetivos da revisão. Um total de 7.540 registros foi identificado e examinado, e 47 artigos foram incluídos. Os domínios cognitivos mais frequentemente relatados como alterados de 4 a 12 semanas após a COVID-19 aguda foram linguagem, memória episódica e função executiva e, após 12 semanas, os domínios mais afetados foram atenção, memória episódica e função executiva. Os resultados dessa revisão de escopo destacam que adultos com síndrome pós-aguda da COVID-19 podem apresentar comprometimento em domínios cognitivos específicos.

## INTRODUCTION

Since the beginning of the COVID-19 pandemic, the number of studies reporting persistent symptoms after the acute phase of infection has increased, especially those showing changes in cognition. 1 Previous longitudinal studies are limited by unharmonized assessments and heterogeneity in follow-up periods, age groups, severity of infection, and symptoms investigated. 1

Different definitions have been used to describe the sequelae after the acute phase of COVID-19. 2 3 Nalbandian et al. 4 classified post-acute COVID-19 syndrome into two categories:

- subacute or ongoing symptomatic COVID-19, which includes symptoms and abnormalities present from 4 to 12 weeks after the onset of infection, and
- chronic or post-COVID-19 syndrome, which includes symptoms and abnormalities persisting beyond 12 weeks.

A retrospective observational study by Mao et al., 5 conducted at the beginning of the pandemic, concluded that 36.4% of infected patients had neurologic manifestations, particularly individuals aged >50 years and with more severe COVID-19 symptoms. A systematic review conducted by Lopez-Leon et al. 6 found that 80% of the patients who recovered from COVID-19 had one or more symptoms (fatigue, hair loss, and dyspnea) beyond the acute phase. The most prevalent neurological symptoms were headache (44%), attention deficit (27%), olfactory disorders (21%), and memory loss (16%).

Although COVID-19 manifests itself mainly as a respiratory infection, it can affect multiple organs, 7 including the brain. At least four possible pathogenic mechanisms may account for the detrimental effects of COVID-19 on the Central Nervous System (CNS):

- direct viral encephalitis,
- systemic inflammation,
- peripheral organ dysfunction (liver, kidney, lung), and
- cerebrovascular changes (stroke, ischemia). 8 The neurological manifestations of COVID-19 may arise from a combination of these factors or from other yet unknown mechanisms.

COVID-19 may aggravate a pre-existing neurological disorder or initiate a new disorder. 8 9 A previous study, using structural MRI and diffuse tensor imaging (DTI), investigated the possible micro-structural changes in the CNS of 60 patients who had recovered from COVID-19 after hospitalization, and in 39 individuals not infected by the disease, and concluded that changes in white matter integrity may explain long-term neurological consequences. 10 Another study detected cortical atrophy in mildly infected individuals, together with cognitive dysfunction (particularly dysexecutive), suggesting that the virus negatively impacts the CNS, regardless of the severity of the initial infection. 11 Previous studies have shown that encephalopathies associated with the virus can lead to cognitive impairments or trigger the development of dementia, 12 a pattern previously observed in earlier epidemics involving the coronavirus. 13

Two recent systematic reviews investigating cognitive changes associated with post-acute COVID-19 syndrome have been found. 1 14 In a review, Tavares-Júnior et al. 14 assessed cognitive impairment related to COVID-19 infection 12 weeks or less after the onset of the infection, and after 12 weeks, to differentiate acute and sub-acute cognitive sequelae. The authors reported that, in 25 studies, the most frequently affected domains were executive function, attention, and episodic memory. A systematic review and meta-analysis conducted by Crivelli et al. 1 evaluated cognitive deficits in COVID-19 patients who previously exhibited no cognitive impairment. The results were interpreted according to the acute and post-COVID-19 phases of the disease and suggested that memory, executive functions, and attention were most frequently impaired. In addition, the meta-analysis results indicated that post-COVID-19 patients had lower Montreal Cognitive Assessment (MoCA) scores than the non-infected controls. The present scoping review advances the understanding of the effect of post-acute COVID-19 syndrome on cognition by focusing exclusively on post-acute COVID-19 syndrome studies, excluding those with data regarding the acute phase. In addition, the severity of infection was considered in the interpretation of the findings, as it is plausible to suppose that disease severity may modulate the long-term effects of COVID-19 on cognition.

The objective of the present review was to examine studies reporting cognitive impairment in post-acute COVID-19 syndrome according to the two categories defined by Nalbandian et al. 4

## METHODS

The present scoping review was conducted according to JBI© methodology for scoping reviews. 15 The Preferred Reporting Items for Systematic Reviews and Meta-Analyses for Scoping Reviews (PRISMA-ScR) 16 extension was used to guide knowledge synthesis. This review was performed according to a protocol previously registered in the Open Science Framework that followed the JBI© methodology (blinded to reviewers). According to the *JBI Manual for Evidence Synthesis* , 16 experts in practice and research on cognitive impairment and COVID-19 were consulted when preparing the protocol and when discussing the results of the present scoping review.

### Review question

‘Which cognitive domains may be impaired in adults with post-COVID-19 syndrome?”

### Inclusion criteria

#### *Participants*

The review included studies on individuals aged ≥18 years who had cognitive impairment in post-acute COVID-19 syndrome. In addition, results were classified according to the severity of the infection (mild without hospitalization, mild with hospitalization (without ICU or intubation), and severe with need for ICU and/or intubation). Complete mapping and description of all evidence were performed for both sexes, and only human studies were included. Studies involving children (under 18 years of age) or those in which it was difficult to single out adults were excluded.

#### *Concept*

The concept of cognitive dysfunction elected for this review is related to neurological causes. Cognitive dysfunction encompasses deficits caused by a range of neurological disorders, including direct viral encephalitis, systemic inflammation, peripheral organ dysfunction (liver, kidneys, and lungs), and cerebrovascular problems. Therefore, this scoping review investigated cognitive impairment (i.e., deficits in episodic memory, attention, language, executive, and visuospatial functions) secondary to COVID-19 manifesting from four weeks after infection.

For the purpose of this review, post-acute COVID-19 syndrome was defined as persistent symptoms and/or delayed or long-term complications of infection beyond four weeks from the onset of symptoms. These symptoms persist owing to an inflammatory or viral host response that occurs approximately 4 weeks after the initial infection and continues for a period. 5 Based on recent literature, 4 it is further divided into two categories:

- subacute or ongoing symptomatic COVID-19, which includes symptoms and abnormalities present from 4 to 12 weeks after the onset of infection, and
- chronic or post-COVID-19 syndrome, which includes symptoms and abnormalities persisting beyond 12 weeks.

#### *Context*

This scoping review covered studies examining the impacts of COVID-19 on cognition in individuals with subacute, chronic, or post-acute COVID-19 syndrome treated in inpatient, outpatient, rehabilitation settings, and home care.

#### *Types of study*

The studies included in this review were observational, clinical/experimental trials, case studies, and reports. Publications were excluded if they failed to meet the selection criteria established; were published in languages other than those accepted for the present study (English, Spanish, or Portuguese); were published in the form of abstracts in congress annals; study protocols; editorials; theses and dissertations; discussions or reports; addressed techniques for improving cognition (cognitive stimulation, cognitive training, treatment methods, etc.); involved the effects of lockdown and social distancing on cognition; and studies involving individuals with a previous diagnosis of mental disorders, dementia, or prior cognitive impairments. Articles not peer-reviewed or grey literature were not included in the present review. Searches were restricted to studies published from 2019 onwards due to the acceptance of the virus. 3

#### *Search strategy*

A 3-step search strategy was employed in this review. A limited initial search of PubMed was conducted, followed by the analysis of titles and abstracts, along with the indexing terms used to describe articles. A second search was performed with all search words and index terms identified for all databases with a search period from December 2019 to June 2021 and with two updates in January 2022 and December 2022. A manual search was conducted for additional empirical studies. Our search terms were derived from our initial searches and were: “Cognitive Dysfunction” AND (“Coronavirus Infections” OR “COVID-19”) AND Pandemics). However, this strategy was applied individually to each database, as described in Supplementary Material (available at https://www.arquivosdeneuropsiquiatria.org/wp-content/uploads/2023/09/ANP-2023.8004-Supplementary-Material.docx ).

Only studies published in English, Portuguese, or Spanish were included in the review because time and resource constraints precluded the inclusion of papers and other resources written in other languages.

The databases that were searched included PubMed, PubMed PMC, BVS – BIREME, Embase, SCOPUS, Cochrane, Web of Science, Proquest, PsycInfo, and EBSCOHost.

#### *Study selection*

EndNote X8 (Clarivate Analytics, PA, USA) was used to manage the records and remove duplicates. We used Rayyan ^®^ where articles were blindly reviewed by two independent reviewers (GCC and VA). Two independent reviewers selected the titles and abstracts for assessment according to the review inclusion and exclusion criteria. Articles that met the inclusion criteria were retrieved and analyzed by two independent reviewers (GCC and VA) based on the inclusion and exclusion criteria. If the two independent reviewers did not agree, a third reviewer (RCM) was consulted.

#### *Data extraction*

Data were extracted using Microsoft Excel, including details on the study reference, country of origin, context, participant characteristics (age, sex), study design, description of the type of care or clinical setting of participants (treated in inpatient, outpatient, rehabilitation settings, and home care), infection severity, post-acute COVID-19 syndrome, and key findings relevant to the objective of the review.

#### *Data analysis and presentation*

The selected literature was mapped in terms of quantity, type, characteristics, and sources of evidence, according to the objective of this scoping review. According to the guidelines of the *JBI Manual for Evidence Synthesis* , 15 the mapping process of this stage involved the extraction of data provided by the abstracts of each article (n = 47) for author, year, title, country, objective and methodology, population and methods, summary of findings, key messages, and limitations, as presented in Table 1 . Two authors (GCC and VA) extracted and mapped the data for the studies in the data extraction table, while a third author (RCM) checked the extracted data.

**Table 1:** Population characteristics and context of studies included in the scoping review (N = 47).

Author (year)	City, Country	Type	Setting	N	Average age (SD)	Female n (%)	Assessment of dysphoric symptoms	Tool of assessment
Frontera et al., 52 (2022)	New York, USA	Longitudinal prospective	Hospital	129	65 (-)	48 (37.2)	Yes	MoCA
Miskowiak et al., 49 (2022)	Bispebjerg, Denmark	Longitudinal	Hospital	25	56 (10.7)	12 (48.0)	Yes	Screen for Cognitive Impairment in Psychiatry Danish Version; Trail Making Test Part B; Cognitive Failure Questionnaire
Crivelli et al., 18 (2022)	Buenos Aires, Argentina	Cross- Sectional	Outpatient	29	50 (43.6)	22 (49.0)	Yes	Argentine version of the MoCA; Trail Making A; Digit Span Forwards; Digit-Symbol Coding; Craft Story 21; Rey Auditory Verbal Learning Test Delayed Recall from the Benson Figure Test; Trail Making B; Wisconsin Card Sorting Test; Stroop Test; Phonological fluency; Benson Figure; Clock Drawing Test; Multilingual Naming Test; Semantic fluency.
Pémille et al., 54 (2022)	Paris, France	Longitudinal prospective	Hospital	13	62 (-)	8 (61.5)	Yes	MMSE; Frontal Assessment Battery; 40 words oral naming test for language; Dubois five words test; Forwards and or Backwards Digit Spans; Similarities test of the Wechsler Adult Intelligence Scale 4th edition; Brixton test; Stroop Color-Word Test-Victoria version; categorical and lexical verbal fluencies.
Cristillo et al., 19 (2022)	Brescia, Italy	Cross- Sectional	Hospital	25	68.5 (11.1)	7 (29.2)	Yes	MoCA
Larsson et al., 55 (2022)	Uppsala, Suécia	Longitudinal prospective	Hospital	46	59 (-)	5 (28.0)	Yes	MoCA
Kim et al., 56 (2021)	Daegu, South Korea	Longitudinal prospective	Hospital	241	–	77 (32.0)	Yes	Self-report for cognitive impairment (loss of intellectual functions such as thinking, remembering, and reasoning), difficulty concentrating, amnesia (memory loss and inability to recall facts, information and experiences)
Holdsworth et al., 35 (2022)	United Kingdom, England	Longitudinal	Community	205	38.3 (-)	34 (16.6)	Yes	The NIH Toolbox of Neurological and Behavioral Function.
Braga et al., 36 (2022)	Brasilia, Brazil	Longitudinal	Hospital and Outpatient	614	47.6 (11.2)	451 (73.0)	Yes	The Barrow Neurological Institute Screen for Higher Cerebral Functions; NEUPSILIN; Clock Drawing Test
Carrillo-Garcia et al., 37 (2022)	Madrid, Spain	Longitudinal	Hospital	165	88.5 (6.73)	114 (69.1)	Yes	Red Cross Mental
Cecchetti et al., 38 (2022)	Milan, Italy	Longitudinal	Outpatient	33	60.6 (12.9)	8 (24.2)	Yes	Tests investigating the global cognition, executive functions, memory, visuospatial functions, language.
Ferrucci et al., 39 (2022)	Milan, Italy	Longitudinal	Hospital	76	56.24 (12.1)	20 (26.3)	Yes	MoCA; Brief Repeatable Battery of Neuropsychological Tests; The Serial Recall Test; The Spatial Recall Test; The Symbol-Digit Modalities Test; The Paced Serial Additions Test; Word List Generation
Hadad et al., 40 (2022)	Haifa, Israel	Longitudinal	Community	46	49.5 (11.5)	30.0 (65.0)	No	MoCA
Jaquet et al., 57 (2022)	Paris, France	Longitudinal prospective	Hospital	41	56.0 (-)	10.0 (24.0)	Yes	MoCA
Liu et al., 58 (2022)	Wuhan, China	Longitudinal prospective	Hospital	1.438	–	747 (51.9)	No	Telephone Interview of Cognitive Status-40; The Informant Questionnaire on Cognitive Decline in the Elderly
Mattioli et al., 59 (2021)	Brescia, Italy	Longitudinal prospective	Hospital	215	–	135 (62.8)	No	MMSE; Cognitive Impairment Index; Controlled Oral Word Association; Rey figure copy and recall; California Verbal Learning Test; Rey Auditory Verbal Learning Test
Nersesjan et al., 62 (2022)	Copenhagen, Denmark	Prospective case-control	Hospital	85	56.8 (14.0)	36 (42.0)	Yes	MoCA
Ollila et al., 63 (2022)	Helsinki, Finland	Prospective controlled cohort	Hospital	165	–	91 (55.1)	Yes	Wechsler Adult Intelligence Scale-IV coding; Continuous Performance Test; Stroop Naming; Trail Making B; Stroop Interference; Frontal Assessment Battery; Wechsler Memory Scale version III; Rey Complex Figure
Stavem et al., 41 (2022)	Norway	Longitudinal	Hospital	233	50.1 (14.8)	137 (59.0)	Yes	Cambridge Neuropsychological Test Automated Battery; Motor screening test; Delayed matching to sample; One-touch Stockings of Cambridge; Rapid visual information processing; Spatial working memory.
Vannorsdall et al., 42 (2021)	Baltimore, USA	Longitudinal	Outpatient	82	54.5 (14.6)	48 (58.5)	Yes	Rey Auditory Verbal Learning Test; Oral Trail Making Test parts A and B; Number span task; Letter-cued verbal fluency; Category-cued verbal fluency.
Kay et al., 20 (2022)	Boston, Baltimore, Charlestown, USA and Aachen, Germany	Cross-sectional	Outpatient	84	–	49 (58.3)	No	Comprehensive neuropsychological testing of each city.
Priftis et al., 21 (2022)	Pádua, Italy	Cross-sectional	Rehabilitation Unit	22	58 (-)	5 (22.7)	No	MMSE; Complex Rey figure (copy and delayed recall); Corsi forward and backward; Digit span forward and backward; Rey Auditory Verbal Learning test; Semantic fluency; Trail making test; Stroop test; Wisconsin Card sorting test; Weigl's sorting test; Digit Symbol Modalities test; Phonemic fluency6
Hartung et al., 60 (2022)	Germany	Prospective multicentre	Hospital	969	–	535 (55.0)	Yes	MoCA
Del Brutto et al., 52 (2022)	Atahualpa, Ecuador	Longitudinal	Community	78	62.7 (11.3)	49 (62.8)	Yes	MoCA
Delgado-Alonso et al., 22 (2022)	Madrid, Spain	Cross- Sectional	Hospital	100	51.0 (11.6)	37 (37.0)	Yes	Battery Vienna Test System®
Zhao et al., 23 (2022)	London, England	Cross- Sectional	Community	136	28.6 (9.7)	54 (39.7)	Yes	Cognitive Failures Questionnaire
Latronico et al., 61 (2022)	Brescia, Italy	Longitudinal prospective	Hospital	114	–	26 (22.8)	Yes	MoCA
Bonizzato et al., 44 (2022)	Mantua, Italy	Longitidinal	Rehabilitation Unit	12	71.33 (10.0)	5 (41.7)	Yes	MMSE and MoCA
Pilotto et al., 45 (2021)	Brescia, Italy	Longitudinal	Hospital	165	64.8 (12.6)	50 (30.3)	No	MoCA
Albu et al., 24 (2021)	Barcelona, Spain	Cross-Sectional	Rehabilitation Unit	30	54 (-)	11 (36.6)	Yes	Barcelona Test; Orientation Test Rey Auditory Verbal; Learning Test; Digit Span Backward subtest of the Wechsler Adult Intelligence Scale III; PMR task Number Span Backward
Alemanno et al., 46 (2021)	Milan, Italy	Longitudinal	Rehabilitation Unit	87	67.2 (12.9)	25 (28.7)	Yes	MMSE and MoCA
Becker et al., 25 (2021)	New York, USA	Cross- Sectional	Outpatient, Emergency and Hospital	740	49 (14.2)	464 (62.7)	No	Digit Span Forward and Backward; Trail Making Test Part A and B; Phonemic and Category Verbal Fluency; Hopkins Verbal Learning Test– Revised.
Carrillo-Garcia et al., 47 (2021)	Madrid, Spain	Longitudinal	Hospital	165	88.5 (6.7)	114 (69.1)	No	Red Cross Mental Scale
Dressing et al., 48 (2022)	Freiburg, Germany	Longitudinal	Hospital	31	54 (2.0)	20 (64.5)	No	Hopkins Verbal Learning Test-Revised; Brief Visuospatial Memory Test-Revised; Digit Span Forward and Backward; Trail Making Test Part A and B; Color-Word Interference Test; Symbol-Digit Modalities Test; Semantic and Letter Fluency Test
Hosp et al., 26 (2021)	Freiburg, Germany	Cross- Sectional	Hospital	29	65.2 (14.4)	11 (37.9)	No	MoCA
Lamontagne et al., 27 (2021)	Canada e USA	Cross- Sectional	Community	50	30.8 (7.7)	29 (58.0)	Yes	Attention Network Test
Liu et al., 28 (2021)	Wuhan, China	Cross- Sectional	Hospital	1,539	69 (-)	801 (52.0)	No	Chinese version of the Telephone; Interview of Cognitive Status-40; Informant Questionnaire on Cognitive Decline in the Elderly.
Miskowiak et al., 34 (2021)	Bispebjerg, Denmark	Longitudinal	Hospital	29	56.2 (10.6)	12 (41.3)	No	Screen for Cognitive Impairment in Psychiatry Danish Version; Trail Making Test Part B; Cognitive Failure Questionnaire
Pistarin et al., 29 (2021)	Milan, Italy	Cross- Sectional	Rehabilitation Unit	40	64.1 (11.8)	25 (62.5)	Yes	MMSE and MoCA
Weidman et al., 50 (2022)	New York, USA	Longitudinal	Hospital	87	62 (-)	64 (73.5)	Yes	MoCA
Blazhenets et al., 51 (2021)	Freiburg, Germany	Longitudinal	Hospital	8	66 (14.2)	2 (25.0)	No	MoCA
Evans et al., 30 (2021)	England, Northern Ireland, Scotland e Wales	Cross- Sectional	Hospital	1.077	58.0 (13.0)	384 (35,7)	No	MoCA
Graham et al., 7 (2021)	Chicago, USA	Cross- Sectional	Hospital	100	43.2 (1.3)	70 (70.0)	No	NIH Toolbox v2
Hampshire et al., 31 (2020)	United Kingdom, England	Cross- Sectional	Community	81.337	46.7 (15.7)	44.826 (55.1)	No	Block Rearrange; Tower of London; Digit Span; Spatial Span; Target Detection; 2D Mental Rotation Test; Analogical Reasoning
Del Brutto et al., 43 (2021)	Atahualpa, Ecuador	Longitudinal	Community	93	62.6 (11.0)	59 (63.4)	Yes	MoCA
Almeria et al., 32 (2020)	Barcelona, Spain	Cross- Sectional	Hospital	35	47.6 (8.9)	19 (54.2)	Yes	Espanha Complutense Verbal Learning Test; Visual Reproduction of the Wechsler Memory Scale –IV; Digit Span Forward and Backward, Letter and Numbers; Trail Making Test Part A and B; Symbol Digit Modalities Test; STROOP Test; Phonemic and Semantic Verbal Fluency; Boston Naming Test from the NEURONORMA project.
Woo et al., 33 (2020)	Hamburg, Germany	Cross- Sectional	Hospital	18	42.2 (14.3)	10 (55.5)	Yes	TICS-M

Abbreviations: MMSE, Mini Mental Status Examination; MoCA, Montreal Cognitive Assessment; NIH, National Institutes of Health Toolbox v2.1 instrument; TICS-M, Modified Telephone Interview for Cognitive Status.

## RESULTS

### Literature search

The initial search of all the databases was performed in June 2021, with two further updates in January and December 2022. The search strategy was designed to be sensitive and consequently led to the retrieval of a large number of studies. The database search identified 7,540 records, of which 4,114 were excluded as duplicates, generating a total of 3,426 studies for the reading of titles and abstracts. Of these studies, 3,316 were subsequently excluded because they did not meet the inclusion criteria; 110 records were analyzed, with three later excluded because the full text could not be accessed. A total of 107 studies were eligible for full reading, of which 66 were excluded after applying the inclusion criteria.

**Figure 1:**
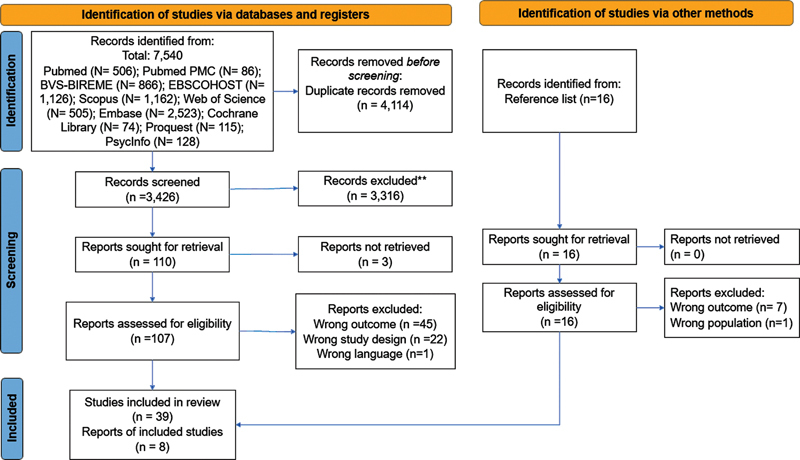
Study selection process for inclusion in the review.

A total of 39 studies were included in the review. In addition to these studies, other sources were manually searched, yielding an additional eight eligible records. Therefore, a total of 47 studies were included in this review. The PRISMA 17 flow diagram depicted in Figure 1 shows the study selection process for inclusion in the review.

### Publication dates and study types included

Of the 47 studies included, four were published in 2020, 17 in 2021, and 26 in 2022. Of the total, 17 studies had a cross-sectional design, 7 18 19 20 21 22 23 24 25 26 27 28 29 30 31 32 33 19 34 35 36 37 38 39 40 41 42 43 44 45 46 47 48 49 50 51 52 had a longitudinal design, nine prospective longitudinal designs, 53 54 55 56 57 58 59 60 61 one prospective case-control 62 and one prospective controlled cohort design 63 ( Table 1 ).

### Countries and regions

All 47 studies reviewed were in English, comprising 32 from Europe, seven from the USA, four from Asia, four from South America, and one from Canada. However, to further elucidate studies by country, these regions were categorized into high, upper-middle-income, high-middle, middle-low, and low-income countries (LMIC) according to the World Bank classification in the 2020–2021 fiscal year. Based on this stratification, 41 studies were carried out in high-income countries, and six in upper-middle-income. Table 1 presents the breakdown by country.

### Population characteristics

The characteristics of the populations investigated in these studies are summarized in Table 1 . Most studies (n = 33) focused on the population aged ≥ 18 years. Three studies included individuals aged ≥ 40 years. 21 43 64 Individuals aged ≥ 60 years comprised the samples investigated in five studies, 28 37 44 47 58 whereas two studies established no age constraints. 19 27 Regarding the age profile of the reviewed studies, the lowest mean age was 28.6 years 23 and the highest was 88.5 years. 37 47 The sample size ranged from 8 51 to 81,337 31 participants. The studies included men and women; however, the samples were predominantly female in most investigations.

Most studies drew on primary data (n = 39), and seven studies used data from an ongoing study. 35 42 43 53 60 63 64 Participants were recruited from a variety of settings. Most studies were recruited from within the hospital setting (n = 30), five recruited individuals from a rehabilitation setting, six from the community, and one recruited patients from different settings (outpatient, hospital, and emergency room). In a study by Evans et al. 30 collected data from 53 units of the National Health Service (NHS), and Kay et al. 20 collected data from three hospitals in the USA and one hospital in Germany. Hampshire et al. 31 and Zhao et al. 23 recruited participants from the community through online tests. The specific information is provided in Table 1 .

### Disease severity: respiratory treatment types

Of the 47 studies, 20 reported the respiratory treatment that the participants received, but not all, related to the type of respiratory treatment administered with cognitive sequelae. For instance, Alemanno et al. 46 reported that the orotracheal intubation and ventilation groups had higher scores than the oxygen therapy group for executive functions, naming, short- and long-term memory, abstraction, and orientation. However, Almeria et al. 32 reported that the group that required oxygen therapy had lower memory, attention, and executive subtest scores than the asymptomatic group. A recent study investigated patients receiving treatment in the ICU with invasive ventilation–3-6 months after discharge. The authors found that the domain most affected was episodic memory in the delayed recall. 57 In contrast, Liu et al. 28 contradicted the data of Almeria et al., 32 who stated that high-flow oxygen therapy during the acute phase of COVID-19 can alleviate oxygen deficiency and protect against post-infection cognitive decline. A plausible explanation for this disparity may be the difference in the evaluation time of patients after recovery, as worse scores may be associated with oxygen deficiency during the acute stage of the disease.

Only three studies classified the samples into mild symptomatic infection, no infection, and asymptomatic infection. Eight studies reported that some patients needed noninvasive mechanical ventilation, 11 reported the use of oxygen support, and 12 studies reported the use of invasive mechanical ventilation (e.g., orotracheal intubation, tracheostomy, and extracorporeal membrane oxygenation). The study by Alemanno et al. 46 identified participants requiring respiratory assistance and, of the 87 participants, 31 needed orotracheal intubation and ventilation, 18 non-invasive ventilation, 29 oxygen therapy using masks, and only nine needed no oxygen. The studies were grouped according to the severity of infection. Patients receiving oxygen therapy were classified as having mild infection, those receiving non-invasive mechanical ventilation as moderate, and those receiving invasive mechanical ventilation as severe. The findings of the studies are summarized in Table 2 according to post-acute COVID-19 syndrome, respiratory treatment type, severity level (mild, moderate, severe), and cognitive functions affected.

**Table 2:** Persistent symptoms, and disease severity reported in the included studies.

Author (year)	Post-acute COVID-19 syndrome*	Length of stay in the hospital (days)	Severity (n) *(types of tratament)*	Severity rating	Main results
Frontera et al., 52 (2022)	6 and 12 months	**-**	Intubation Worts SOFA score Lowest oxygen saturation	Severe Light Light	Below-normal MoCA scores were observed in 50% of patients without cognitive impairment, regardless of the presence or absence of a neurological complication during hospitalization. But with improvement at six- and 12-month follow-up.
Miskowiak et al., 49 (2022)	12 months	**-**	**-**	**-**	Cognitive impairments were seen after 1 year in half of patients hospitalized with COVID-19, but the cognitive sequelae were stable over time from three months to one year after hospitalization.
Crivelli et al., 18 (2022)	average of 142 days	**-**	**-**	**-**	The results show that deficits can be identified predominantly in executive functions and attention and have a smaller effect on memory and language in outpatients who have had COVID-19.
Pémille et al., 54 (2022)	3 months	**-**	Intubation O2 Support	Severe Ligth	At baseline the results showed severe acute cognitive dysfunction with abnormal scores on the global MMSE test, affecting mainly executive functions and episodic memory. All patients improved between baseline and follow-up evaluations.
Cristillo et al., 19 (2022)	12 months	**-**	**-**	**-**	Patients who reported cognitive deficits (n = 25) showed a decline in MoCA after one year of discharge.
Larsson et al., 55 (2022)	4 and 12 months	23	Invasive ventilation therapy	Severe	The results showed no improvements between the first and second follow-up.
Kim et al., 56 (2021)	12 months	**-**	**-**	**-**	Overall, 52.7% responders still experienced COVID-19-related persistent symptoms. The main symptoms were difficulty in concentration, cognitive dysfunction, amnesia, depression, fatigue, and anxiety
Holdsworth et al., 35 (2022)	> 3 months	**-**	**-**	**-**	69% reported ≥ 3 ongoing symptoms. Shortness of breath (61%), fatigue (54%) and cognitive problems (47%) were the most frequent symptoms, 17% met criteria for anxiety and 24% depression.
Braga et al., 36 (2022)	Average 8 months	**-**	Oxygen support Orotracheal Intubation	Ligth Severe	The results showed that previously hospitalized and non-hospitalized COVID-19 survivors had cognitive deficits, but a relevant difference for disease severity.
Carrillo-Garcia et al., 37 (2022)	6 months	**-**	**-**	**-**	Of the survivors at 6 months, more than half of the sample had some of the following sequelae: dyspnea 20%, functional impairment 41.7%, cognitive impairment 31.3% or depressive symptoms 42.4%.
Cecchetti et al., 38 (2022)	10 months	**-**	**-**	**-**	At follow-up, 36% of patients showed an impairment in at least one cognitive domain. 3%, 6% and 6% of patients showed an executive, memory and visual-spatial impairment, respectively, and 21% of subjects showed a multidomain impairment.
Ferrucci et al., 39 (2022)	5 and 12 months	12	Oxygen support	Ligth	Compared to the assessment at 5 months, verbal memory, attention, and processing speed improved significantly after 1 year, whereas visuospatial memory did not. The most affected domains after 1 year were processing speed, long-term visuospatial and verbal memory.
Hadad et al., 40 (2022)	7 months	**-**	Oxygen support	Light	On the MoCA test, executive functions, particularly phonemic fluency, and attention, were impaired. In contrast, the total MoCA score, and memory and orientation sub scores did not differ from expected ranges
Jaquet et al., 57 (2022)	3 and 6 months	36	Invasive mechanical ventilation	Severe	MOCA was 26 (23–28.5), and cognitive impairment was reported in 17 patients. The most affected domain was delayed recall with a score of 4 (2–4) in a scale of 0–5.
Liu et al., 58 (2022)	6 and 12 months	**-**	**-**	**-**	The incidence of cognitive impairment in survivors 12 months after discharge was 12.45%. Severe COVID-19 was associated with a higher risk of early-onset cognitive decline, late-onset cognitive decline, and progressive cognitive decline, while no severe COVID-19 was associated with a higher risk of early-onset cognitive decline
Mattioli et al., 59 (2021)	4 months	**-**	Continuous Positive Airway Pressure Mechanical ventilation O2 support	Light Light Light	MMSE resulted within normal limits in all patients, with a statistically significant lower score in ICU patients and the raw mean scores of all the neuropsychological tests resulted significantly lower in ICU than in non-ICU patients.
Nersesjan et al., 62 (2022)	6 months	**-**	**-**	**-**	The cognitive status improved substantially, from 19.2 (95% CI, 15.2-23.2) at discharge to 26.1 (95% CI, 23.1-29.1) for 15 patients with COVID-19 with MoCA evaluations from hospital discharge.
Ollila et al., 63 (2022)	6 months	20	Invasive mechanical ventilation	Severe	The total cognitive score at six months post-COVID differed between the groups (Home group, Hospitalized non-ICU group (WARD) and ICU group). In pairwise comparisons, both ICU and WARD patients performed worse than home group.
Stavem et al., 41 (2022)	8 to 13 months	**-**	**-**	**-**	The proportion of respondents with z-scores lower than -1.5 was similarly small, though with larger effects in post hoc analyses of executive function among older respondents.
Vannorsdall et al., 42 (2021)	4 months	**-**	**-**	**-**	Cognitive deficits were widespread in those with and without ICU stays and occurred most on measures of oral processing speed and verbal fluency as well as learning and memory. Patients requiring at least 48 hours of ICU care demonstrated poorer global cognition and in the executive functioning and working memory.
Kay et al., 20 (2022)	average 7 months	**-**	**-**	**-**	Cognitive deficits were in processing speed, followed by executive functions and attention/working memory; there was more variability in findings about memory (encoding and delayed memory) and language/semantic access domains across sites.
Priftis et al., 21 (2022)	average 2 months	**-**	Tracheostomy Artificial ventilation	Severe Severe	None of the patients showed impaired performance on measures assessing overall cognitive status, visuo-spatial short-term/working memory, and language production (semantic fluency).
Hartung et al., 60 (2022)	6 months	**-**	**-**	**-**	26% of patients had mild and 1% had moderate cognitive impairment
Del Brutto et al., 52 (2022)	6 and 18 months	–	Mild symptomatic infections No infection	Light Negative	The post-pandemic cognitive decline seen after 6 months occurred primarily in individuals who had COVID-19. After 18 months, the difference in the total MoCA score was not significant between the groups.
Delgado-Alonso et al., 22 (2022)	> 9 months	average of 19	Ventilatory assistance	Light	COVID-19 patients showed decreased performance on tests of attention and executive function, processing speed, working memory, and inhibition; episodic memory; and visuospatial processing.
Zhao et al., 23 (2022)	> 4 months	–	–	–	COVID-19 survivors performed well on most of the cognitive skills tested, including working memory, executive function, planning and mental rotation. They showed changes in episodic memory tests (up to 6 months after infection) and surveillance (up to 9 months).
Latronico et al., 61 (2022)	3, 6 and 12 months	**-**	Mechanical Ventilation Tracheostomy	Severe Severe	During the evaluations, the prevalence of cognitive deficit in the MoCA exam decreased. At three months there were 23 patients and at 12 months there were seven.
Bonizzato et al., 44 (2022)	3 months	**-**	**-**	**-**	No significant differences were found over time (T0, T1 and T2) to the screening test, but between T0 and T1, the mean scores at MoCA showed a slight difference.
Pilotto et al., 45 (2021)	6 months	average of 11.6	Oxygen support Non-invasive ventilation Intubation	Ligth Moderate Severe	At neurological examination, 40% of patients exhibited neurological abnormalities, such as hyposmia (18.0%), cognitive deficits (17.5%), postural tremor (13.8%) and subtle motor/sensory deficits
Albu et al., 24 (2021)	> 3 months	average of 26	O2 Support Non-invasive ventilation Invasive ventilation	Light Moderate Severe	In patients who received respiratory assistance, persistent cognitive deficits (difficulties in concentration, short-term memory impairment) occurred after recovery. However, there was no difference between the group that did not receive assistance.
Alemanno et al., 46 (2021)	1 month	80	Orotracheal intubation and ventilation Non-invasive ventilation Oxygen therapy with masks Did not need oxygen	Severe Moderate Moderate Light	The orotracheal intubation and ventilation group scored higher than the oxygen therapy group on tests of executive functions, naming, short- and long-term memory, abstraction, and orientation.
Becker et al., 25 (2021)	> 7 months	–	–	–	Hospitalized patients were more likely to have deficits in attention, executive functioning, categorical verbal fluency, and episodic memory than those in the outpatient group. Patients treated in the emergency department were more likely to have impaired categorical verbal fluency and memory than those treated in the outpatient clinic.
Carrillo-Garcia et al., 47 (2021)	3 months	15	–	–	The results showed that among the survivors, two out of three patients continued to have physical disability, cognitive impairment or affective complaints or anorexia.
Dressing et al., 48 (2022)	> 3 months	–	–	–	Patients who had COVID-19 performed above normal in all cognitive domains (verbal memory, visual memory, processing speed, attention, executive function) and on the total MoCA score.
Hosp et al., 26 (2021)	1 month	–	Only observation Non-invasive ventilation Endotracheal ventilation	Light Moderate Severe	MoCA performance was altered in 18/26 patients (mean score 21.8/30) with greater impairment in frontoparietal cognitive functions
Lamontagne et al., 27 (2021)	> 4 months	–	Asymptomatic Mild symptomatic infections	Asymptomatic Light	Individuals with mild symptomatic infections (post-COVID-19) had impairment in executive functioning but not in attentional orientation or alertness, highlighting the specificity of post-infection cognitive dysfunction.
Liu et al., 28 (2021)	6 months	–	Mechanical ventilation High Flow Oxygen Therapy	Moderate Severe	COVID-19 patients had worse cognitive performance 6 months after recovery. In addition, high-flow oxygen therapy during the acute phase of COVID-19, which can alleviate oxygen deficiency, may protect against post-infection cognitive decline.
Miskowiak et al., 34 (2021)	3-4 months	–	–	–	The percentage of patients with clinically significant cognitive impairment ranged from 59% to 65%, depending on the cutoff used, with verbal learning and executive functions being the most affected.
Pistarin et al., 29 (2021)	3 months	–	–	–	The post-covid-19 group, and COVID-19 patients showed deficits in executive function, short- and long-term memory, visuospatial skills, abstraction, and orientation. However, post-COVID-19 patients, one month after infection, performed better in the language subdomain, compared to COVID-19 patients.
Weidman et al., 50 (2022)	1 month	51	Intubation Mechanical ventilation	Moderate Severe	In total, 25% of post-ICU patients had cognitive impairment. However, there were no associations between length of ICU stay, delirium, exposure to benzodiazepines, steroids, or systemic paralytics with positive screening for physical, psychological, or cognitive impairment.
Blazhenets et al., 51 (2021)	> 3 months	–	–	–	A significant improvement in MoCA was observed, relative to the control group, but the average performance was within the mild cognitive impairment range established with the normative data.
Evans et al., 30 (2021)	2-7 months	Class 3- 4: 2 Class 5: 6 Class 6: 10 Class 7-9: 33	class 3–4: no need for continuous supplemental oxygen class 5: continuous supplemental oxygen only class 6: ventilation with continuous positive airway pressure, bilevel positive airway pressure, or high flow nasal oxygen class 7–9: invasive mechanical ventilation or extracorporeal membrane oxygenation	Light Light Moderate Severe	Four clusters were identified with different severity of mental and physical health impairment (n = 767): very severe (131 patients, 17%), severe (159, 21%), moderate together with cognitive impairment (127, 17%) and mild (350, 46%).
Graham et al., 7 (2021)	> 1 month (mean 5.27)	–	–	–	Study data showed that SARS-CoV-2 patients performed worse on cognitive attention and working memory tasks compared to a demographically matched US population.
Hampshire et al., 31 (2020)	> 3 months	–	No disease Asymptomatic No home assistance Home assistance Hospitalized without ventilation Hospitalized with ventilation	No disease Asymptomatic Light Light Moderate Moderate	Data showed that cognitive deficits were of large and moderate effect size for people who were hospitalized (N = 192), but also for non-hospitalized cases who had biological confirmation of COVID-19 infection (N = 326).
Del Brutto et al., 43 (2021)	6 months	–	Mild symptomatic infections No infection	Light Negative	Individuals with a history of mild symptomatic SARS-CoV-2 infections are more than 18 times more likely to develop cognitive decline than those without clinical and serological evidence of infection. .
Almeria et al., 32 (2020)	1 month	average of 25	Oxygen Asymptomatic	Light Asymptomatic	Overall, 34.3% of patients had cognitive complaints after COVID-19 infection, and those who required oxygen therapy had lower scores on the memory, attention, and executive function subtests compared to asymptomatic patients.
Woo et al., 33 (2020)	20–105 days (median, 85 days)	–	Oxygen	Light	78% of patients reported mild cognitive deficits and performed worse on tests of short-term memory, attention, and concentration compared to 10 healthy age-matched controls. However, cognitive outcomes did not correlate with hospitalization, treatment, viremia, or acute inflammation.

Note: * According to Nalbadian et al. 5 it is further divided into two categories: (1) subacute or ongoing symptomatic covid-19, which includes symptoms and abnormalities present 4–12weeks after acute covid-19; and (2) chronic or post-covid-19 syndrome, which includes symptoms and abnormalities persisting or present beyond 12 weeks of the onset of acute covid-19.

### Assessment of persistent cognitive symptoms

All studies assessed the participants' cognition after the acute phase using validated cognitive tests. Persistent cognitive symptoms were classified according to the two post-acute COVID-19 phases proposed by Nalbandian et al. 4

Of the 47 studies reviewed, 12 assessed the cognition of participants 4–12 weeks after infection, and 35 assessed cognitions beyond 12 weeks of the onset of COVID-19 ( Table 2 ). Most notably, in the study by Zhao et al., 23 longitudinal observations showed that episodic memory declined for up to six months post-infection, and monitoring of time during a memory task declined for up to nine months. In contrast, Blazhenets et al. 51 evaluated patients three months after the onset of COVID-19 and found that chronic individuals showed a significant improvement on a cognitive screening test (MoCA) compared to controls, but mean performance remained within the mild cognitive impairment range. del Brutto et al., 43 six months after the onset of acute COVID-19, showed possible recovery from persistent cognitive symptoms in the post-acute COVID-19 syndrome, but another study 48 showed persistent cognitive deficits after three months. Additionally, Latronico et al. 61 investigated patients three, six, and 12 months after infection and found that the prevalence of cognitive impairment in the MoCA decreased consecutively. Another longitudinal study found no improvement in cognition after seven months of infection. 40 Further information is presented in Table 2 .

### Decline in cognitive domains in post-acute COVID-19 syndrome

The present review mapped the possible cognitive sequelae in patients infected with SARS-CoV-2, with most studies confirming the hypothesis that impairments in specific cognitive functions persist after infection. We have separated these findings according to the phases defined by Nalbandian et al. 4

### 4-12 weeks of the onset of acute COVID-19

In this category, we found 12 articles that met the eligibility criteria. Alemanno et al. 46 evaluated patients in the subacute phase (one month after infection) and found that patients who went to the ICU and required some type of respiratory support (for example, orotracheal intubation and ventilation) scored higher than the oxygen therapy group in tests of executive functions, naming, short- and long-term memory, abstraction, and orientation. In another study, Weidman et al. 50 found that of the patients who stayed in the ICU, 25% had cognitive impairments. Additionally, Almeria et al. 32 observed that patients using oxygen had lower memory, attention, and executive function subtest scores than asymptomatic patients.

Nine articles assessed cognitive domains separately, while three 44 46 50 studies employed scales measuring global cognition in their analyses, such as the MoCA and MMSE, reporting only total scores on these instruments and precluding classification of changes into specific cognitive domains.

**Figure 2:**
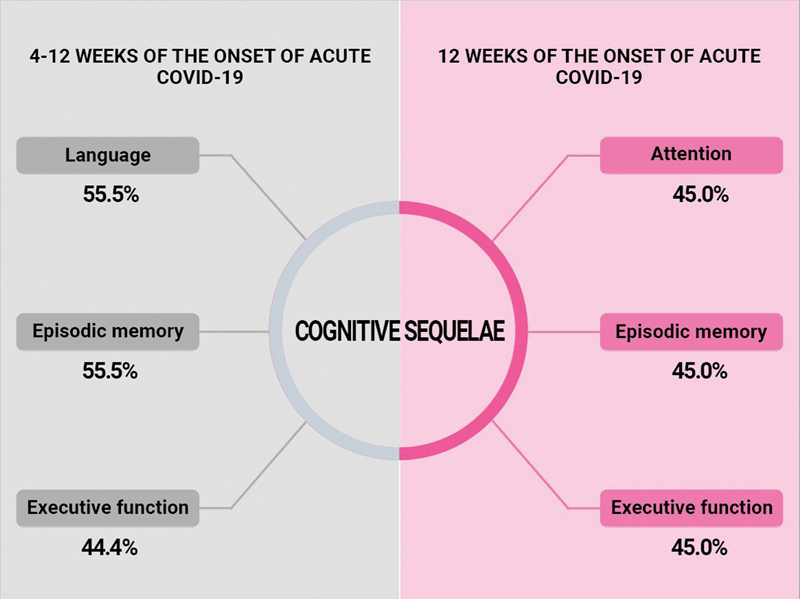
Frequency of the main cognitive alterations according to the categories proposed by Nalbandian et al. 5

The cognitive aspects most frequently evaluated were executive function (n = 8), episodic memory (n = 6), attention (n = 7), language (n = 5), abstraction (n = 4), and global cognition (n = 3). The most frequently reported cognitive domains were language (55.5%), attention (55.5%), episodic memory (44.4%), and executive functions (33.3%). More specific information can be found in Figure 2 .

### 12 weeks of the onset of acute COVID-19

In this category, we identified 35 articles that met the eligibility criteria. For instance, Albu et al. 24 reported that patients in the post-ICU and non-ICU subgroups had cognitive and affective changes (attention difficulties, altered concentration, impaired short-term memory, and anxiety) after infection with COVID-19. In addition, the study found that 62.5% of the patients admitted to the ICU had neurological complications. Becker et al. 25 showed that hospitalized patients were more prone to experience deficits in attention, executive function, and episodic memory after COVID-19 infection compared to outpatients. In Liu et al., 28 both severe and non-severe patients had cognitive impairment six months after infection, particularly severe cases, and 35.71% presented cognitive deficits. Another study found that COVID-19 patients had lower performance than healthy controls on tests evaluating attention, executive function, working memory, episodic memory, and visuospatial processing. 22

In this category, 20 articles evaluated cognitive domains separately, whereas 15 studies used scales that measure global cognition, such as the MoCA, NIH Toolbox, RCM, TICS-40, and MMSE. And eight showed a global decline.

However, Mattioli et al., 59 Ferrucci et al., 39 and Hadad et al. 40 separated cognitive domains for more specific assessments. The cognitive domains most frequently evaluated in the studies were executive function (n = 15), attention (n = 13), episodic memory (n = 12), and working memory (n = 10). The domains most frequently reported as altered were attention (45.0%), episodic memory (45.0%), executive function (45.0%), and processing speed (20.0%). More specific information can be found in Figure 2 .

## DISCUSSION

The objective of this scoping review was to map and assess the existing literature on cognitive sequelae in post-acute COVID-19 syndrome. Overall, the results of the included studies showed that there was an impact on cognitive functions after infection.

The present review was able to map the cognitive domains that showed a decline in the two phases of the post-acute syndrome of COVID-19 (4 to 12 weeks after infection and after 12 weeks), indicating that despite the time elapsed after infection, cognitive sequelae could still be observed. In fact, of the 37 studies that evaluated cognition in the post-acute COVID-19 syndrome, deficits in memory and executive functions were observed in both phases; however, language impairment was more prevalent in the post-acute phase and attention in the chronic phase.

Most studies included in the present review used the MoCA ( Table 1 ) instead of the MMSE as a global cognitive screening test. Alemanno et al. 46 and Pfoh et al. 65 noted that the MoCA evaluation proved more sensitive than the MMSE for detecting cognitive deficits among patients who tended to perform worse in this task when compared to non-infected controls.

Regarding disease severity, some studies showed that individuals undergoing mechanical ventilation (invasive or not) had worse cognitive performance compared to patients who did not need such treatments, 26 28 46 while others failed to find this association. 24 27 One study reported that hospitalized patients had greater impairment of executive function, attention, and memory than those who did not require hospital admission, 25 suggesting that disease severity is indeed associated with worse cognitive impairment, a conclusion supported by Jaywant et al. 66 However, Miskowiak et al. 34 found no association between the disease severity and cognitive function. Previous studies have suggested that disease severity is associated with worse cognitive deficits.

The studies included in this scoping review noted that some cognitive domains affected by COVID-19 had improved after a certain amount of time. For example, del Brutto et al. 52 documented cognitive deficits at six months from disease onset, with improvement after one year. Another study also reported cognitive impairments in adult patients one year after disease diagnosis and suggested that a longer presence of cognitive deficits may be related to patient autoimmunity. 33 Latronico et al. 59 reported that during the evaluations (3, 6, and 12 months), the prevalence of cognitive deficit in the MoCA exam decreased. At three months there were 23 patients, and at 12 months there were seven who had deficits in the MoCA exam.

Previous studies have reported that cognitive impairment in post-acute COVID-19 syndrome may be associated with risk factors, such as older age, low educational level, premorbid comorbidities, delirium, male sex, and history of neuropsychiatric disease. 23 60 Regarding age, five studies in this review included older adults. 28 37 44 47 58 Studies with samples of older adults suggested there is a high percentage of cognitive impairment in this group - 60% three months after the infection 47 and 30% after six months. 37 The incidence of cognitive impairment 12 months after discharge was 12.45%. 58 These findings suggest that cognitive changes in post-acute COVID-19 are of particular concern among older patients, even more so, if they were severely infected. 58

It is noteworthy that most studies are from high-income countries, indicating a pressing need for more investigations exploring the issue of COVID-19 sequelae in middle-to-low-income countries such as Brazil, a nation that has been seriously impacted by the disease. It is vital to investigate the COVID-19 aftermath in regions where diagnostic and care options are less accessible.

This review has limitations. First, no search of international gray literature was conducted, given the need to disseminate results to health professionals. Performing a more in-depth search of international gray literature might have helped reduce publication bias. The absence of this source may also have led to the non-inclusion of some relevant studies, but endeavors were made to review the reference lists of citations for additional studies. Another limitation is that the reviewed studies failed to include information on pre-infection cognitive status, precluding comparisons with pre-illness conditions. A strength of the present scoping review is its clear focus on post-acute COVID-19 syndrome and its effort to analyze the impact of disease severity on reported cognitive deficits.

In conclusion, the studies reviewed indicated that cognitive deficits were present in the subacute and chronic phases of post-acute COVID-19 syndrome, particularly in episodic memory and executive functions. Some of the reviewed studies have reported an association between the severity of the disease and long-term cognitive deficits. Monitoring cognitive sequelae after acute SARS-CoV-2 infection can help implement rehabilitation protocols. Intervention studies based on cognitive rehabilitation may provide an evidence-based treatment to address symptoms that are frequent and affect everyday performance. Most importantly, studies should explore the influence of different socioeconomic situations on the cognition of infected individuals and their recovery, considering factors such as income, education, and access to healthcare.
