# Supplementary material for: Cognitive impairment in post-acute COVID-19 syndrome: a scoping review
Source: Arq Neuropsiquiatr. 2023 Dec 29;81(12):1053–69. doi: 10.1055/s-0043-1777115 (PMC10756850; doi:10.1055/s-0043-1777115)
Supplement: Supplementary file 1 — Supplementary Material [file 10-1055-s-0043-1777115-s238004.pdf]

SUPPLEMENTARY MATERIAL

Search strategy

Source	Strategy	Total	Update January 2022	Update December 2022
**PUBMED**	((Cognitive Dysfunction[MeSH Terms]) OR ("Cognitive Dysfunction"[Title/Abstract] OR "Cognitive Dysfunctions"[-Title/Abstract] OR "Dysfunction, Cognitive"[Title/Abstract] OR "Dysfunctions, Cognitive"[Title/Abstract] OR "Cognitive Impairments"[Title/Abstract] OR "Cognitive Impairment"[-Title/Abstract] OR "Impairment, Cognitive"[Title/Abstract] OR "Impairments, Cognitive"[Title/Abstract] OR "Mild Cognitive Impairment"[Title/Abstract] OR "Cognitive Impairment, Mild"[-Title/Abstract] OR "Cognitive Impairments, Mild"[-Title/Abstract] OR "Impairment, Mild Cognitive"[Title/Abstract] OR "Impairments, Mild Cognitive"[Title/Abstract] OR "Mild Cognitive Impairments"[Title/Abstract] OR "Mild Neurocognitive Disorder"[Title/Abstract] OR "Disorder, Mild Neurocognitive"[Title/Abstract] OR "Disorders, Mild Neurocognitive"[Title/Abstract] OR "Mild Neurocognitive Disorders"[Title/Abstract] OR "Neurocognitive Disorder, Mild"[Title/Abstract] OR "Neurocognitive Disorders, Mild"[-Title/Abstract] OR "Cognitive Decline"[Title/Abstract] OR "Cognitive Declines"[Title/Abstract] OR "Decline, Cognitive"[-Title/Abstract] OR "Declines, Cognitive"[Title/Abstract] OR "Mental Deterioration"[Title/Abstract] OR "Deterioration, Mental"[Title/Abstract] OR "Deteriorations, Mental"[-Title/Abstract] OR "Mental Deteriorations"[Title/Abstract])) AND (((((((Coronavirus Infections[MeSH Terms]) OR ("Coronavirus Infections"[Title/Abstract] OR "Coronavirus Infection"[-Title/Abstract] OR "Infection, Coronavirus"[Title/Abstract] OR "Infections, Coronavirus"[Title/Abstract] OR "Middle East Respiratory Syndrome"[Title/Abstract] OR "MERS (Middle East Respiratory Syndrome)"[Title/Abstract])) OR ("COVID-19"[-Title/Abstract] OR "2019 novel coronavirus disease"[-Title/Abstract] OR "COVID19"[Title/Abstract] OR "COVID-19 pandemic"[Title/Abstract] OR "SARS-CoV-2 infection"[-Title/Abstract] OR "COVID-19 virus disease"[Title/Abstract] OR "2019 novel coronavirus infection"[Title/Abstract] OR "2019-nCoV infection"[Title/Abstract] OR "coronavirus disease 2019"[Title/Abstract] OR "coronavirus disease-19"[-Title/Abstract] OR "2019-nCoV disease"[Title/Abstract] OR "COVID-19 virus infection"[Title/Abstract])) OR ((COVID-19"[MeSH Terms]) OR ("COVID-19"[Title/Abstract] OR "COVID 19"[Title/Abstract] OR "COVID-19 Virus Disease"[Title/Abstract] OR "COVID 19 Virus Disease"[Title/Abstract] OR "COVID-19 Virus Diseases"[Title/Abstract] OR "Disease, COVID-19 Virus"[-Title/Abstract] OR "Virus Disease, COVID-19"[Title/Abstract] OR "COVID-19 Virus Infection"[Title/Abstract] OR "COVID 19 Virus Infection"[Title/Abstract] OR "COVID-19 Virus Infections"[-Title/Abstract] OR "Infection, COVID-19 Virus"[Title/Abstract] OR "Virus Infection, COVID-19"[Title/Abstract] OR "2019-nCoV Infection"[Title/Abstract] OR "2019 nCoV Infection"[-Title/Abstract] OR "2019-nCoV Infections"[Title/Abstract] OR "Infection, 2019-nCoV"[Title/Abstract] OR "Coronavirus Disease-19"[Title/Abstract] OR "Coronavirus Disease 19"[-Title/Abstract] OR "2019 Novel Coronavirus Disease"[Title/Abstract] OR "2019 Novel Coronavirus Infection"[Title/Abstract] OR "2019-nCoV Disease"[-Title/Abstract] OR "2019 nCoV Disease"[Title/Abstract] OR "2019-nCoV Diseases"[Title/Abstract] OR "Disease, 2019-nCoV"[Title/Abstract] OR COVID19[Title/Abstract] OR "Coronavirus Disease 2019"[Title/Abstract] OR "Disease 2019, Coronavirus"[Title/Abstract] OR "SARS Coronavirus 2 Infection"[Title/Abstract] OR "SARS-CoV-2 Infection"[-Title/Abstract] OR "Infection, SARS-CoV-2"[Title/Abstract] OR	187	298	506

 © 2023. The Author(s).

(*Continued*)

Source	Strategy	Total	Update January 2022	Update December 2022
	"SARS CoV 2 Infection"[Title/Abstract] OR "SARS-CoV-2 Infections"[Title/Abstract] OR "COVID-19 Pandemic"[-Title/Abstract] OR "COVID 19 Pandemic"[Title/Abstract] OR "COVID-19 Pandemics"[Title/Abstract] OR "Pandemic, COVID-19"[Title/Abstract]))) OR ((COVID-19"[MeSH Terms]) OR ("COVID-19"[Title/Abstract] OR "COVID 19"[Title/Abstract] OR "COVID-19 Virus Disease"[Title/Abstract] OR "COVID 19 Virus Disease"[Title/Abstract] OR "COVID-19 Virus Diseases"[-Title/Abstract] OR "Disease, COVID-19 Virus"[Title/Abstract] OR "Virus Disease, COVID-19"[Title/Abstract] OR "COVID-19 Virus Infection"[Title/Abstract] OR "COVID 19 Virus Infection"[-Title/Abstract] OR "COVID-19 Virus Infections"[Title/Abstract] OR "Infection, COVID-19 Virus"[Title/Abstract] OR "Virus Infection, COVID-19"[Title/Abstract] OR "2019-nCoV Infection"[-Title/Abstract] OR "2019 nCoV Infection"[Title/Abstract] OR "2019-nCoV Infections"[Title/Abstract] OR "Infection, 2019-nCoV"[Title/Abstract] OR "Coronavirus Disease-19"[-Title/Abstract] OR "Coronavirus Disease 19"[Title/Abstract] OR "2019 Novel Coronavirus Disease"[Title/Abstract] OR "2019 Novel Coronavirus Infection"[Title/Abstract] OR "2019-nCoV Disease"[Title/Abstract] OR "2019 nCoV Disease"[-Title/Abstract] OR "2019-nCoV Diseases"[Title/Abstract] OR "Disease, 2019-nCoV"[Title/Abstract] OR COVID19[-Title/Abstract] OR "Coronavirus Disease 2019"[Title/Abstract] OR "Disease 2019, Coronavirus"[Title/Abstract] OR "SARS Coronavirus 2 Infection"[Title/Abstract] OR "SARS-CoV-2 Infection"[Title/Abstract] OR "Infection, SARS-CoV-2"[-Title/Abstract] OR "SARS CoV 2 Infection"[Title/Abstract] OR "SARS-CoV-2 Infections"[Title/Abstract] OR "COVID-19 Pandemic"[Title/Abstract] OR "COVID 19 Pandemic"[-Title/Abstract] OR "COVID-19 Pandemics"[Title/Abstract] OR "Pandemic, COVID-19"[Title/Abstract]))) OR ("SARS-CoV"[-Title/Abstract] OR "SARS-CoV2"[Title/Abstract] OR "2019-nCoV disease"[Title/Abstract] OR "2019-nCoV infection"[-Title/Abstract] OR "COVID 19"[Title/Abstract] OR "COVID 2019"[Title/Abstract] OR "nCoV 2019 disease"[Title/Abstract] OR "nCoV 2019 infection"[Title/Abstract] OR "novel coronavirus 2019 disease"[Title/Abstract] OR "novel coronavirus 2019 infection"[Title/Abstract] OR "novel coronavirus disease 2019"[Title/Abstract] OR "novel coronavirus infection 2019"[-Title/Abstract] OR "Wuhan coronavirus disease"[Title/Abstract] OR "Wuhan coronavirus infection"[Title/Abstract])) AND ((Pandemics) OR (Pandemics[Title/Abstract] OR Pandemic [Title/Abstract])))			
**PUBMED PMC**	(("cognitive defect") OR ((Cognitive Dysfunction[MeSH Terms]) OR ("Cognitive Dysfunction"[Title/Abstract] OR "Cognitive Dysfunctions"[Title/Abstract] OR "Dysfunction, Cognitive"[-Title/Abstract] OR "Dysfunctions, Cognitive"[Title/Abstract] OR "Cognitive Impairments"[Title/Abstract] OR "Cognitive Impairment"[Title/Abstract] OR "Impairment, Cognitive"[-Title/Abstract] OR "Impairments, Cognitive"[Title/Abstract] OR "Mild Cognitive Impairment"[Title/Abstract] OR "Cognitive Impairment, Mild"[Title/Abstract] OR "Cognitive Impairments, Mild"[Title/Abstract] OR "Impairment, Mild Cognitive"[-Title/Abstract] OR "Impairments, Mild Cognitive"[-Title/Abstract] OR "Mild Cognitive Impairments"[Title/Abstract] OR "Mild Neurocognitive Disorder"[Title/Abstract] OR "Disorder, Mild Neurocognitive"[Title/Abstract] OR "Disorders, Mild Neurocognitive"[Title/Abstract] OR "Mild Neurocognitive Disorders"[Title/Abstract] OR "Neurocognitive Disorder, Mild"[Title/Abstract] OR "Neurocognitive Disorders, Mild"[-Title/Abstract] OR "Cognitive Decline"[Title/Abstract] OR	32	45	86

(*Continued*)

 © 2023. The Author(s).

(*Continued*)

Source	Strategy	Total	Update January 2022	Update December 2022
	"Cognitive Declines"[Title/Abstract] OR "Decline, Cognitive"[-Title/Abstract] OR "Declines, Cognitive"[Title/Abstract] OR "Mental Deterioration"[Title/Abstract] OR "Deterioration, Mental"[Title/Abstract] OR "Deteriorations, Mental"[-Title/Abstract] OR "Mental Deteriorations"[Title/Abstract]))) AND ((((("Coronavirus Infections"[MeSH Terms]) OR ("Coronavirus Infections"[Title/Abstract] OR "Coronavirus Infection"[-Title/Abstract] OR "Infection, Coronavirus"[Title/Abstract] OR "Infections, Coronavirus"[Title/Abstract] OR "Middle East Respiratory Syndrome"[Title/Abstract] OR "MERS (Middle East Respiratory Syndrome)"[Title/Abstract])) OR ("COVID-19"[-Title/Abstract] OR "2019 novel coronavirus disease"[-Title/Abstract] OR "COVID19"[Title/Abstract] OR "COVID-19 pandemic"[Title/Abstract] OR "SARS-CoV-2 infection"[-Title/Abstract] OR "COVID-19 virus disease"[Title/Abstract] OR "2019 novel coronavirus infection"[Title/Abstract] OR "2019-nCoV infection"[Title/Abstract] OR "coronavirus disease 2019"[Title/Abstract] OR "coronavirus disease-19"[-Title/Abstract] OR "2019-nCoV disease"[Title/Abstract] OR "COVID-19 virus infection"[Title/Abstract])) OR ((COVID-19 [MeSH Terms]) OR ("COVID-19"[Title/Abstract] OR "COVID 19"[Title/Abstract] OR "COVID-19 Virus Disease"[Title/Abstract] OR "COVID 19 Virus Disease"[Title/Abstract] OR "COVID-19 Virus Diseases"[Title/Abstract] OR "Disease, COVID-19 Virus"[-Title/Abstract] OR "Virus Disease, COVID-19"[Title/Abstract] OR "COVID-19 Virus Infection"[Title/Abstract] OR "COVID 19 Virus Infection"[Title/Abstract] OR "COVID-19 Virus Infections"[-Title/Abstract] OR "Infection, COVID-19 Virus"[Title/Abstract] OR "Virus Infection, COVID-19"[Title/Abstract] OR "2019-nCoV Infection"[Title/Abstract] OR "2019 nCoV Infection"[-Title/Abstract] OR "2019-nCoV Infections"[Title/Abstract] OR "Infection, 2019-nCoV"[Title/Abstract] OR "Coronavirus Disease-19"[Title/Abstract] OR "Coronavirus Disease 19"[-Title/Abstract] OR "2019 Novel Coronavirus Disease"[Title/Abstract] OR "2019 Novel Coronavirus Infection"[Title/Abstract] OR "2019-nCoV Disease"[-Title/Abstract] OR "2019 nCoV Disease"[Title/Abstract] OR "2019-nCoV Diseases"[Title/Abstract] OR "Disease, 2019-nCoV"[Title/Abstract] OR COVID19[Title/Abstract] OR "Coronavirus Disease 2019"[Title/Abstract] OR "Disease 2019, Coronavirus"[Title/Abstract] OR "SARS Coronavirus 2 Infection"[Title/Abstract] OR "SARS-CoV-2 Infection"[-Title/Abstract] OR "Infection, SARS-CoV-2"[Title/Abstract] OR "SARS CoV 2 Infection"[Title/Abstract] OR "SARS-CoV-2 Infections"[Title/Abstract] OR "COVID-19 Pandemic"[-Title/Abstract] OR "COVID 19 Pandemic"[Title/Abstract] OR "COVID-19 Pandemics"[Title/Abstract] OR "Pandemic, COVID-19"[Title/Abstract]))) OR ("SARS-CoV"[Title/Abstract] OR "SARS-CoV2"[Title/Abstract] OR "2019-nCoV disease"[-Title/Abstract] OR "2019-nCoV infection"[Title/Abstract] OR "COVID 19"[Title/Abstract] OR "COVID 2019"[Title/Abstract] OR "nCoV 2019 disease"[Title/Abstract] OR "nCoV 2019 infection"[Title/Abstract] OR "novel coronavirus 2019 disease"[Title/Abstract] OR "novel coronavirus 2019 infection"[Title/Abstract] OR "novel coronavirus disease 2019"[Title/Abstract] OR "novel coronavirus infection 2019"[-Title/Abstract] OR "Wuhan coronavirus disease"[Title/Abstract] OR "Wuhan coronavirus infection"[Title/Abstract])) AND ((Pandemics[MeSH Terms]) OR (Pandemics[Title/Abstract] OR Pandemic[Title/Abstract])))			
**BVS – BIREME**	(("Cognitive Dysfunction" OR "Cognitive Dysfunctions" OR "Dysfunction, Cognitive" OR "Dysfunctions, Cognitive" OR	177	428	866

 © 2023. The Author(s).

(*Continued*)

Source	Strategy	Total	Update January 2022	Update December 2022
	"Cognitive Impairments" OR "Cognitive Impairment" OR "Impairment, Cognitive" OR "Impairments, Cognitive" OR "Mild Cognitive Impairment" OR "Cognitive Impairment, Mild" OR "Cognitive Impairments, Mild" OR "Impairment, Mild Cognitive" OR "Impairments, Mild Cognitive" OR "Mild Cognitive Impairments" OR "Mild Neurocognitive Disorder" OR "Disorder, Mild Neurocognitive" OR "Disorders, Mild Neurocognitive" OR "Mild Neurocognitive Disorders" OR "Neurocognitive Disorder, Mild" OR "Neurocognitive Disorders, Mild" OR "Cognitive Decline" OR "Cognitive Declines" OR "Decline, Cognitive" OR "Declines, Cognitive" OR "Mental Deterioration" OR "Deterioration, Mental" OR "Deteriorations, Mental" OR "Mental Deteriorations") OR ("cognitive defects")) AND ((("Coronavirus Infections" OR "Coronavirus Infection" OR "Infection, Coronavirus" OR "Infections, Coronavirus" OR "Middle East Respiratory Syndrome" OR "MERS (Middle East Respiratory Syndrome)") OR ("COVID-19" OR "2019 novel coronavirus disease" OR "COVID19" OR "COVID-19 pandemic" OR "SARS-CoV-2 infection" OR "COVID-19 virus disease" OR "2019 novel coronavirus infection" OR "2019-nCoV infection" OR "coronavirus disease 2019" OR "coronavirus disease-19" OR "2019-nCoV disease" OR "COVID-19 virus infection") OR ("COVID-19" OR "COVID 19" OR "COVID-19 Virus Disease" OR "COVID 19 Virus Disease" OR "COVID-19 Virus Diseases" OR "Disease, COVID-19 Virus" OR "Virus Disease, COVID-19" OR "COVID-19 Virus Infection" OR "COVID 19 Virus Infection" OR "COVID-19 Virus Infections" OR "Infection, COVID-19 Virus" OR "Virus Infection, COVID-19" OR "2019-nCoV Infection" OR "2019 nCoV Infection" OR "2019-nCoV Infections" OR "Infection, 2019-nCoV" OR "Coronavirus Disease-19" OR "Coronavirus Disease 19" OR "2019 Novel Coronavirus Disease" OR "2019 Novel Coronavirus Infection" OR "2019-nCoV Disease" OR "2019 nCoV Disease" OR "2019-nCoV Diseases" OR "Disease, 2019-nCoV" OR covid19 OR "Coronavirus Disease 2019" OR "Disease 2019, Coronavirus" OR "SARS Coronavirus 2 Infection" OR "SARS-CoV-2 Infection" OR "Infection, SARS-CoV-2" OR "SARS CoV 2 Infection" OR "SARS-CoV-2 Infections" OR "COVID-19 Pandemic" OR "COVID 19 Pandemic" OR "COVID-19 Pandemics" OR "Pandemic, COVID-19") OR ("SARS-CoV" OR "SARS-CoV2" OR "2019-nCoV disease" OR "2019-nCoV infection" OR "COVID 19" OR "COVID 2019" OR "nCoV 2019 disease" OR "nCoV 2019 infection" OR "novel coronavirus 2019 disease" OR "novel coronavirus 2019 infection" OR "novel coronavirus disease 2019" OR "novel coronavirus infection 2019" OR "Wuhan coronavirus disease" OR "Wuhan coronavirus infection")) AND (pandemics OR pandemic))			
**EBSCOHOST**	(("Cognitive Dysfunction" OR "Cognitive Dysfunctions" OR "Dysfunction, Cognitive" OR "Dysfunctions, Cognitive" OR "Cognitive Impairments" OR "Cognitive Impairment" OR "Impairment, Cognitive" OR "Impairments, Cognitive" OR "Mild Cognitive Impairment" OR "Cognitive Impairment, Mild" OR "Cognitive Impairments, Mild" OR "Impairment, Mild Cognitive" OR "Impairments, Mild Cognitive" OR "Mild Cognitive Impairments" OR "Mild Neurocognitive Disorder" OR "Disorder, Mild Neurocognitive" OR "Disorders, Mild Neurocognitive" OR "Mild Neurocognitive Disorders" OR "Neurocognitive Disorder, Mild" OR "Neurocognitive Disorders, Mild" OR "Cognitive Decline" OR "Cognitive Declines" OR "Decline, Cognitive" OR "Declines, Cognitive" OR "Mental Deterioration" OR "Deterioration, Mental" OR "Deteriorations, Mental" OR "Mental Deteriorations") OR ("cognitive defects")) AND ((("Coronavirus Infections" OR "Coronavirus Infection" OR "Infection, Coronavirus" OR "Infections, Coronavirus" OR "Middle East Respiratory Syndrome" OR	322	424	1.126

(*Continued*)

 © 2023. The Author(s).

(*Continued*)

Source	Strategy	Total	Update January 2022	Update December 2022
	"MERS (Middle East Respiratory Syndrome)") OR ("COVID-19" OR "2019 novel coronavirus disease" OR "COVID19" OR "COVID-19 pandemic" OR "SARS-CoV-2 infection" OR "COVID-19 virus disease" OR "2019 novel coronavirus infection" OR "2019-nCoV infection" OR "coronavirus disease 2019" OR "coronavirus disease-19" OR "2019-nCoV disease" OR "COVID-19 virus infection") OR ("COVID-19" OR "COVID 19" OR "COVID-19 Virus Disease" OR "COVID 19 Virus Disease" OR "COVID-19 Virus Diseases" OR "Disease, COVID-19 Virus" OR "Virus Disease, COVID-19" OR "COVID-19 Virus Infection" OR "COVID 19 Virus Infection" OR "COVID-19 Virus Infections" OR "Infection, COVID-19 Virus" OR "Virus Infection, COVID-19" OR "2019-nCoV Infection" OR "2019 nCoV Infection" OR "2019-nCoV Infections" OR "Infection, 2019-nCoV" OR "Coronavirus Disease-19" OR "Coronavirus Disease 19" OR "2019 Novel Coronavirus Disease" OR "2019 Novel Coronavirus Infection" OR "2019-nCoV Disease" OR "2019 nCoV Disease" OR "2019-nCoV Diseases" OR "Disease, 2019-nCoV" OR COVID19 OR "Coronavirus Disease 2019" OR "Disease 2019, Coronavirus" OR "SARS Coronavirus 2 Infection" OR "SARS-CoV-2 Infection" OR "Infection, SARS-CoV-2" OR "SARS CoV 2 Infection" OR "SARS-CoV-2 Infections" OR "COVID-19 Pandemic" OR "COVID 19 Pandemic" OR "COVID-19 Pandemics" OR "Pandemic, COVID-19") OR ("SARS-CoV" OR "SARS-CoV2" OR "2019-nCoV disease" OR "2019-nCoV infection" OR "COVID 19" OR "COVID 2019" OR "nCoV 2019 disease" OR "nCoV 2019 infection" OR "novel coronavirus 2019 disease" OR "novel coronavirus 2019 infection" OR "novel coronavirus disease 2019" OR "novel coronavirus infection 2019" OR "Wuhan coronavirus disease" OR "Wuhan coronavirus infection")) AND (Pandemics OR Pandemic))			
**SCOPUS**	(TITLE-ABS-KEY ("Cognitive Dysfunction" OR "Cognitive Dysfunctions" OR "Dysfunction, Cognitive" OR "Dysfunctions, Cognitive" OR "Cognitive Impairments" OR "Cognitive Impairment" OR "Impairment, Cognitive" OR "Impairments, Cognitive" OR "Mild Cognitive Impairment" OR "Cognitive Impairment, Mild" OR "Cognitive Impairments, Mild" OR "Impairment, Mild Cognitive" OR "Impairments, Mild Cognitive" OR "Mild Cognitive Impairments" OR "Mild Neurocognitive Disorder" OR "Disorder, Mild Neurocognitive" OR "Disorders, Mild Neurocognitive" OR "Mild Neurocognitive Disorders" OR "Neurocognitive Disorder, Mild" OR "Neurocognitive Disorders, Mild" OR "Cognitive Decline" OR "Cognitive Declines" OR "Decline, Cognitive" OR "Declines, Cognitive" OR "Mental Deterioration" OR "Deterioration, Mental" OR "Deteriorations, Mental" OR "Mental Deteriorations") OR ALL ("cognitive defects")) AND ((TITLE-ABS-KEY ("Coronavirus Infections" OR "Coronavirus Infection" OR "Infection, Coronavirus" OR "Infections, Coronavirus" OR "Middle East Respiratory Syndrome" OR "MERS (Middle East Respiratory Syndrome)") OR TITLE-ABS-KEY ("COVID-19" OR "2019 novel coronavirus disease" OR "COVID19" OR "COVID-19 pandemic" OR "SARS-CoV-2 infection" OR "COVID-19 virus disease" OR "2019 novel coronavirus infection" OR "2019-nCoV infection" OR "coronavirus disease 2019" OR "coronavirus disease-19" OR "2019-nCoV disease" OR "COVID-19 virus infection") OR TITLE-ABS-KEY ("COVID-19" OR "COVID 19" OR "COVID-19 Virus Disease" OR "COVID 19 Virus Disease" OR "COVID-19 Virus Diseases" OR "Disease, COVID-19 Virus" OR "Virus Disease, COVID-19" OR "COVID-19 Virus Infection" OR "COVID 19 Virus Infection" OR "COVID-19 Virus Infections" OR "Infection, COVID-19 Virus" OR "Virus Infection, COVID-19" OR "2019-nCoV Infection" OR "2019 nCoV Infection" OR "2019-nCoV Infections" OR "Infection, 2019-nCoV" OR "Coronavirus	396	656	**1.162**

 © 2023. The Author(s).

(*Continued*)

Source	Strategy	Total	Update January 2022	Update December 2022
	Disease-19" OR "Coronavirus Disease 19" OR "2019 Novel Coronavirus Disease" OR "2019 Novel Coronavirus Infection" OR "2019-nCoV Disease" OR "2019 nCoV Disease" OR "2019-nCoV Diseases" OR "Disease, 2019-nCoV" OR covid19 OR "Coronavirus Disease 2019" OR "Disease 2019, Coronavirus" OR "SARS Coronavirus 2 Infection" OR "SARS-CoV-2 Infection" OR "Infection, SARS-CoV-2" OR "SARS CoV 2 Infection" OR "SARS-CoV-2 Infections" OR "COVID-19 Pandemic" OR "COVID 19 Pandemic" OR "COVID-19 Pandemics" OR "Pandemic, COVID-19") OR TITLE-ABS-KEY ("SARS-CoV" OR "SARS-CoV2" OR "2019-nCoV disease" OR "2019-nCoV infection" OR "COVID 19" OR "COVID 2019" OR "nCoV 2019 disease" OR "nCoV 2019 infection" OR "novel coronavirus 2019 disease" OR "novel coronavirus 2019 infection" OR "novel coronavirus disease 2019" OR "novel coronavirus infection 2019" OR "Wuhan coronavirus disease" OR "Wuhan coronavirus infection")) AND (TITLE-ABS-KEY (pandemics OR pandemic)))			
**WEB OF SCIENCE**	TÓPICO: ("Cognitive Dysfunction" OR "Cognitive Dysfunctions" OR "Dysfunction, Cognitive" OR "Dysfunctions, Cognitive" OR "Cognitive Impairments" OR "Cognitive Impairment" OR "Impairment, Cognitive" OR "Impairments, Cognitive" OR "Mild Cognitive Impairment" OR "Cognitive Impairment, Mild" OR "Cognitive Impairments, Mild" OR "Impairment, Mild Cognitive" OR "Impairments, Mild Cognitive" OR "Mild Cognitive Impairments" OR "Mild Neurocognitive Disorder" OR "Disorder, Mild Neurocognitive" OR "Disorders, Mild Neurocognitive" OR "Mild Neurocognitive Disorders" OR "Neurocognitive Disorder, Mild" OR "Neurocognitive Disorders, Mild" OR "Cognitive Decline" OR "Cognitive Declines" OR "Decline, Cognitive" OR "Declines, Cognitive" OR "Mental Deterioration" OR "Deterioration, Mental" OR "Deteriorations, Mental" OR "Mental Deteriorations") OR Todos os campos: ("cognitive defects") Índices = SCI-EXPANDED, SSCI, A&HCI, CPCI-S, CPCI-SSH, ESCI Tempo estipulado = Todos os anos AND TÓPICO: ("Coronavirus Infections" OR "Coronavirus Infection" OR "Infection, Coronavirus" OR "Infections, Coronavirus" OR "Middle East Respiratory Syndrome" OR "MERS (Middle East Respiratory Syndrome) ") OR TÓPICO: ("COVID-19" OR "2019 novel coronavirus disease" OR "COVID19" OR "COVID-19 pandemic" OR "SARS-CoV-2 infection" OR "COVID-19 virus disease" OR "2019 novel coronavirus infection" OR "2019-nCoV infection" OR "coronavirus disease 2019" OR "coronavirus disease-19" OR "2019-nCoV disease" OR "COVID-19 virus infection") OR TÓPICO: ("COVID-19" OR "COVID 19" OR "COVID-19 Virus Disease" OR "COVID 19 Virus Disease" OR "COVID-19 Virus Diseases" OR "Disease, COVID-19 Virus" OR "Virus Disease, COVID-19" OR "COVID-19 Virus Infection" OR "COVID 19 Virus Infection" OR "COVID-19 Virus Infections" OR "Infection, COVID-19 Virus" OR "Virus Infection, COVID-19" OR "2019-nCoV Infection" OR "2019 nCoV Infection" OR "2019-nCoV Infections" OR "Infection, 2019-nCoV" OR "Coronavirus Disease-19" OR "Coronavirus Disease 19" OR "2019 Novel Coronavirus Disease" OR "2019 Novel Coronavirus Infection" OR "2019-nCoV Disease" OR "2019 nCoV Disease" OR "2019-nCoV Diseases" OR "Disease, 2019-nCoV" OR COVID19 OR "Coronavirus Disease 2019" OR "Disease 2019, Coronavirus" OR "SARS Coronavirus 2 Infection" OR "SARS-CoV-2 Infection" OR "Infection, SARS-CoV-2" OR "SARS CoV 2 Infection" OR "SARS-CoV-2 Infections" OR "COVID-19 Pandemic" OR "COVID 19 Pandemic" OR "COVID-19 Pandemics" OR "Pandemic, COVID-19") OR TÓPICO: ("SARS-CoV" OR "SARS-CoV2" OR "2019-nCoV disease" OR "2019-nCoV infection" OR "COVID 19" OR "COVID 2019" OR "nCoV 2019 disease" OR "nCoV 2019	140	267	**505**

(*Continued*)

 © 2023. The Author(s).

(*Continued*)

Source	Strategy	Total	Update January 2022	Update December 2022
	infection” OR “novel coronavirus 2019 disease” OR “novel coronavirus 2019 infection” OR “novel coronavirus disease 2019” OR “novel coronavirus infection 2019” OR “Wuhan coronavirus disease” OR “Wuhan coronavirus infection”) Índices = SCI-EXPANDED, SSCI, A&HCI, CPCI-S, CPCI-SSH, ESCI Tempo estipulado = Todos os anos AND TÓPICO: (Pandemics OR Pandemic) Índices = SCI-EXPANDED, SSCI, A&HCI, CPCI-S, CPCI-SSH, ESCI Tempo estipulado = Todos os anos			
**EMBASE**	’cognitive defect’/syn AND (’coronavirus infection’/syn OR ’coronavirus disease 2019’/syn) AND ’pandemic’/syn	837	1.425	2.523
**COCHRANE LIBRARY**	(“Cognitive Dysfunction” OR “Cognitive Dysfunctions” OR “Dysfunction, Cognitive” OR “Dysfunctions, Cognitive” OR “Cognitive Impairments” OR “Cognitive Impairment” OR “Impairment, Cognitive” OR “Impairments, Cognitive” OR “Mild Cognitive Impairment” OR “Cognitive Impairment, Mild” OR “Cognitive Impairments, Mild” OR “Impairment, Mild Cognitive” OR “Impairments, Mild Cognitive” OR “Mild Cognitive Impairments” OR “Mild Neurocognitive Disorder” OR “Disorder, Mild Neurocognitive” OR “Disorders, Mild Neurocognitive” OR “Mild Neurocognitive Disorders” OR “Neurocognitive Disorder, Mild” OR “Neurocognitive Disorders, Mild” OR “Cognitive Decline” OR “Cognitive Declines” OR “Decline, Cognitive” OR “Declines, Cognitive” OR “Mental Deterioration” OR “Deterioration, Mental” OR “Deteriorations, Mental” OR “Mental Deteriorations”):ti,ab,kw OR (“cognitive defects”) AND (“Coronavirus Infections” OR “Coronavirus Infection” OR “Infection, Coronavirus” OR “Infections, Coronavirus” OR “Middle East Respiratory Syndrome” OR “MERS (Middle East Respiratory Syndrome)”):ti,ab,kw OR (“COVID-19” OR “2019 novel coronavirus disease” OR “COVID19” OR “COVID-19 pandemic” OR “SARS-CoV-2 infection” OR “COVID-19 virus disease” OR “2019 novel coronavirus infection” OR “2019-nCoV infection” OR “coronavirus disease 2019” OR “coronavirus disease-19” OR “2019-nCoV disease” OR “COVID-19 virus infection”):ti,ab,kw OR (“COVID-19” OR “COVID 19” OR “COVID-19 Virus Disease” OR “COVID 19 Virus Disease” OR “COVID-19 Virus Diseases” OR “Disease, COVID-19 Virus” OR “Virus Disease, COVID-19” OR “COVID-19 Virus Infection” OR “COVID 19 Virus Infection” OR “COVID-19 Virus Infections” OR “Infection, COVID-19 Virus” OR “Virus Infection, COVID-19” OR “2019-nCoV Infection” OR “2019 nCoV Infection” OR “2019-nCoV Infections” OR “Infection, 2019-nCoV” OR “Coronavirus Disease-19” OR “Coronavirus Disease 19” OR “2019 Novel Coronavirus Disease” OR “2019 Novel Coronavirus Infection” OR “2019-nCoV Disease” OR “2019 nCoV Disease” OR “2019-nCoV Diseases” OR “Disease, 2019-nCoV” OR COVID19 OR “Coronavirus Disease 2019” OR “Disease 2019, Coronavirus” OR “SARS Coronavirus 2 Infection” OR “SARS-CoV-2 Infection” OR “Infection, SARS-CoV-2” OR “SARS CoV 2 Infection” OR “SARS-CoV-2 Infections” OR “COVID-19 Pandemic” OR “COVID 19 Pandemic” OR “COVID-19 Pandemics” OR “Pandemic, COVID-19”):ti,ab,kw OR (“SARS-CoV” OR “SARS-CoV2” OR “2019-nCoV disease” OR “2019-nCoV infection” OR “COVID 19” OR “COVID 2019” OR “nCoV 2019 disease” OR “nCoV 2019 infection” OR “novel coronavirus 2019 disease” OR “novel coronavirus 2019 infection” OR “novel coronavirus disease 2019” OR “novel coronavirus infection 2019” OR “Wuhan coronavirus disease” OR “Wuhan coronavirus infection”):ti,ab,kw AND (Pandemics OR Pandemic):ti,ab,kw	25	34	74
**PROQUEST**	(su(“Cognitive Dysfunction” OR “Cognitive Dysfunctions” OR “Dysfunction, Cognitive” OR “Dysfunctions, Cognitive” OR “Cognitive Impairments” OR “Cognitive Impairment” OR	18	58	115

 © 2023. The Author(s).

(*Continued*)

Source	Strategy	Total	Update January 2022	Update December 2022
	"Impairment, Cognitive" OR "Impairments, Cognitive" OR "Mild Cognitive Impairment" OR "Cognitive Impairment, Mild" OR "Cognitive Impairments, Mild" OR "Impairment, Mild Cognitive" OR "Impairments, Mild Cognitive" OR "Mild Cognitive Impairments" OR "Mild Neurocognitive Disorder" OR "Disorder, Mild Neurocognitive" OR "Disorders, Mild Neurocognitive" OR "Mild Neurocognitive Disorders" OR "Neurocognitive Disorder, Mild" OR "Neurocognitive Disorders, Mild" OR "Cognitive Decline" OR "Cognitive Declines" OR "Decline, Cognitive" OR "Declines, Cognitive" OR "Mental Deterioration" OR "Deterioration, Mental" OR "Deteriorations, Mental" OR "Mental Deteriorations") OR "cognitive defects") AND ((su("Coronavirus Infections" OR "Coronavirus Infection" OR "Infection, Coronavirus" OR "Infections, Coronavirus" OR "Middle East Respiratory Syndrome" OR "MERS (Middle East Respiratory Syndrome)") OR su("COVID-19" OR "2019 novel coronavirus disease" OR "COVID19" OR "COVID-19 pandemic" OR "SARS-CoV-2 infection" OR "COVID-19 virus disease" OR "2019 novel coronavirus infection" OR "2019-nCoV infection" OR "coronavirus disease 2019" OR "coronavirus disease-19" OR "2019-nCoV disease" OR "COVID-19 virus infection") OR su("COVID-19" OR "COVID 19" OR "COVID-19 Virus Disease" OR "COVID 19 Virus Disease" OR "COVID-19 Virus Diseases" OR "Disease, COVID-19 Virus" OR "Virus Disease, COVID-19" OR "COVID-19 Virus Infection" OR "COVID 19 Virus Infection" OR "COVID-19 Virus Infections" OR "Infection, COVID-19 Virus" OR "Virus Infection, COVID-19" OR "2019-nCoV Infection" OR "2019 nCoV Infection" OR "2019-nCoV Infections" OR "Infection, 2019-nCoV" OR "Coronavirus Disease-19" OR "Coronavirus Disease 19" OR "2019 Novel Coronavirus Disease" OR "2019 Novel Coronavirus Infection" OR "2019-nCoV Disease" OR "2019 nCoV Disease" OR "2019-nCoV Diseases" OR "Disease, 2019-nCoV" OR COVID19 OR "Coronavirus Disease 2019" OR "Disease 2019, Coronavirus" OR "SARS Coronavirus 2 Infection" OR "SARS-CoV-2 Infection" OR "Infection, SARS-CoV-2" OR "SARS CoV 2 Infection" OR "SARS-CoV-2 Infections" OR "COVID-19 Pandemic" OR "COVID 19 Pandemic" OR "COVID-19 Pandemics" OR "Pandemic, COVID-19") OR su("SARS-CoV" OR "SARS-CoV2" OR "2019-nCoV disease" OR "2019-nCoV infection" OR "COVID 19" OR "COVID 2019" OR "nCoV 2019 disease" OR "nCoV 2019 infection" OR "novel coronavirus 2019 disease" OR "novel coronavirus 2019 infection" OR "novel coronavirus disease 2019" OR "novel coronavirus infection 2019" OR "Wuhan coronavirus disease" OR "Wuhan coronavirus infection")) AND su(Pandemics OR Pandemic))			
**PSYCINFO**	((Any Field: ("Cognitive Dysfunction") OR Any Field: ("Cognitive Dysfunctions") OR Any Field: ("Dysfunction, Cognitive") OR Any Field: ("Dysfunctions, Cognitive") OR Any Field: ("Cognitive Impairments") OR Any Field: ("Cognitive Impairment") OR Any Field: ("Impairment, Cognitive") OR Any Field: ("Impairments, Cognitive") OR Any Field: ("Mild Cognitive Impairment") OR Any Field: ("Cognitive Impairment, Mild") OR Any Field: ("Cognitive Impairments, Mild") OR Any Field: ("Impairment, Mild Cognitive") OR Any Field: ("Impairments, Mild Cognitive") OR Any Field: ("Mild Cognitive Impairments") OR Any Field: ("Mild Neurocognitive Disorder") OR Any Field: ("Disorder, Mild Neurocognitive") OR Any Field: ("Disorders, Mild Neurocognitive") OR Any Field: ("Mild Neurocognitive Disorders") OR Any Field: ("Neurocognitive Disorder, Mild") OR Any Field: ("Neurocognitive Disorders, Mild") OR Any Field: ("Cognitive Decline") OR Any Field: ("Cognitive Declines") OR Any Field: ("Decline, Cognitive") OR Any Field: ("Declines, Cognitive") OR Any Field: ("Mental Deterioration") OR Any Field: ("Deterioration, Mental") OR Any	36	69	128

(*Continued*)

 © 2023. The Author(s).

(*Continued*)

Source	Strategy	Total	Update January 2022	Update December 2022
	Field: ("Deteriorations, Mental") OR Any Field: ("Mental Deteriorations")) OR (Any Field: ("cognitive defects"))) AND ((((Any Field: ("Coronavirus Infections")) OR (Any Field: ("Coronavirus Infection")) OR (Any Field: ("Infection, Coronavirus")) OR (Any Field: ("Infections, Coronavirus")) OR (Any Field: ("Middle East Respiratory Syndrome")) OR (Any Field: ("MERS (Middle East Respiratory Syndrome)"))) OR ((Any Field: ("COVID-19")) OR (Any Field: ("2019 novel coronavirus disease")) OR (Any Field: ("COVID19")) OR (Any Field: ("COVID-19 pandemic")) OR (Any Field: ("SARS-CoV-2 infection")) OR (Any Field: ("COVID-19 virus disease")) OR (Any Field: ("2019 novel coronavirus infection")) OR (Any Field: ("2019-nCoV infection")) OR (Any Field: ("coronavirus disease 2019")) OR (Any Field: ("coronavirus disease-19")) OR (Any Field: ("2019-nCoV disease")) OR (Any Field: ("COVID-19 virus infection"))) OR ((Any Field: ("COVID-19")) OR (Any Field: ("COVID 19")) OR (Any Field: ("COVID-19 Virus Disease")) OR (Any Field: ("COVID 19 Virus Disease")) OR (Any Field: ("COVID-19 Virus Diseases")) OR (Any Field: ("Disease, COVID-19 Virus")) OR (Any Field: ("Virus Disease, COVID-19")) OR (Any Field: ("COVID-19 Virus Infection")) OR (Any Field: ("COVID 19 Virus Infection")) OR (Any Field: ("COVID-19 Virus Infections")) OR (Any Field: ("Infection, COVID-19 Virus")) OR (Any Field: ("Virus Infection, COVID-19")) OR (Any Field: ("2019-nCoV Infection")) OR (Any Field: ("2019 nCoV Infection")) OR (Any Field: ("2019-nCoV Infections")) OR (Any Field: ("Infection, 2019-nCoV")) OR (Any Field: ("Coronavirus Disease-19")) OR (Any Field: ("Coronavirus Disease 19")) OR (Any Field: ("2019 Novel Coronavirus Disease")) OR (Any Field: ("2019 Novel Coronavirus Infection")) OR (Any Field: ("2019-nCoV Disease")) OR (Any Field: ("2019 nCoV Disease")) OR (Any Field: ("2019-nCoV Diseases")) OR (Any Field: ("Disease, 2019-nCoV")) OR (Any Field: (COVID19)) OR (Any Field: ("Coronavirus Disease 2019")) OR (Any Field: ("Disease 2019, Coronavirus")) OR (Any Field: ("SARS Coronavirus 2 Infection")) OR (Any Field: ("SARS-CoV-2 Infection")) OR (Any Field: ("Infection, SARS-CoV-2")) OR (Any Field: ("SARS CoV 2 Infection")) OR (Any Field: ("SARS-CoV-2 Infections")) OR (Any Field: ("COVID-19 Pandemic")) OR (Any Field: ("COVID 19 Pandemic")) OR (Any Field: ("COVID-19 Pandemics")) OR (Any Field: ("Pandemic, COVID-19"))) OR ((Any Field: ("SARS-CoV")) OR (Any Field: ("SARS-CoV2")) OR (Any Field: ("2019-nCoV disease")) OR (Any Field: ("2019-nCoV infection")) OR (Any Field: ("COVID 19")) OR (Any Field: ("COVID 2019")) OR (Any Field: ("nCoV 2019 disease")) OR (Any Field: ("nCoV 2019 infection")) OR (Any Field: ("novel coronavirus 2019 disease")) OR (Any Field: ("novel coronavirus 2019 infection")) OR (Any Field: ("novel coronavirus disease 2019")) OR (Any Field: ("novel coronavirus infection 2019")) OR (Any Field: ("Wuhan coronavirus disease")) OR (Any Field: ("Wuhan coronavirus infection")))) AND (((Any Field: (Pandemics)) OR (Any Field: (Pandemic)))))			

 © 2023. The Author(s).
